# Chemical Constituents from *Euphorbia kansui*

**DOI:** 10.3390/molecules22122176

**Published:** 2017-12-08

**Authors:** Qiao Zhang, Qin-Rong Zhou, Jian-Wei Lou, Pei-Dong Chen, Wei-Feng Yao, Wei-Wei Tao, Yu-Ping Tang, Guan-Cheng Dai, Kun Wang, Li Zhang

**Affiliations:** 1 Jiangsu Key Laboratory for High Technology Research of TCM Formulae, National and Local Collaborative Engineering Center of Chinese Medicinal Resources Industrialization and Formulae Innovative Medicine and Jiangsu Collaborative Innovation Center of Chinese Medicinal Resources Industrialization, Nanjing University of Chinese Medicine, Nanjing 210023, China; 18700081184@163.com (Q.Z.); r790507807@gmail.com (Q.-R.Z.); trustlou@163.com (J.-W.L.); chenpeidong1970@163.com (P.-D.C.); yaowf@njucm.edu.cn (W.-F.Y.); tw845@163.com (W.-W.T.); 18260028211@163.com (G.-C.D.); 18260028237@163.com (K.W.); 2 College of Pharmacy and Shaanxi Collaborative Innovation Center of Chinese Medicinal Resources Industrialization, Shaanxi University of Chinese Medicine, Xi’an 712046, China; 2051001@sntcm.edu.cn

**Keywords:** Euphane and Tirucallane, triterpenes, cytotoxicity, *Euphorbia kansui*

## Abstract

In this research, a new triterpenoid, tirucalla-8,24-diene-3β,11β-diol-7-one (**1**), and eupha-8,24-diene-3β,11β-diol-7-one (**2**), which was isolated from *Euphorbia kansui* for the first time, together with twelve other known compounds (**3**–**14**), were isolated from the ethyl acetate extract of *Euphorbia kansui*. Their structures were elucidated based on High resolution electrospray ionization mass spectrometry (HR-ESI-MS), Infrared Spectroscopy (IR), 1D and 2D Nuclear Magnetic Resonance (NMR) data. Both constituents **1** and **2** exhibited moderate cytotoxicity against colon cancer HCT-116, gastric cancer MKN-45 and breast cancer MCF-7.

## 1. Introduction

**Figure 1:**
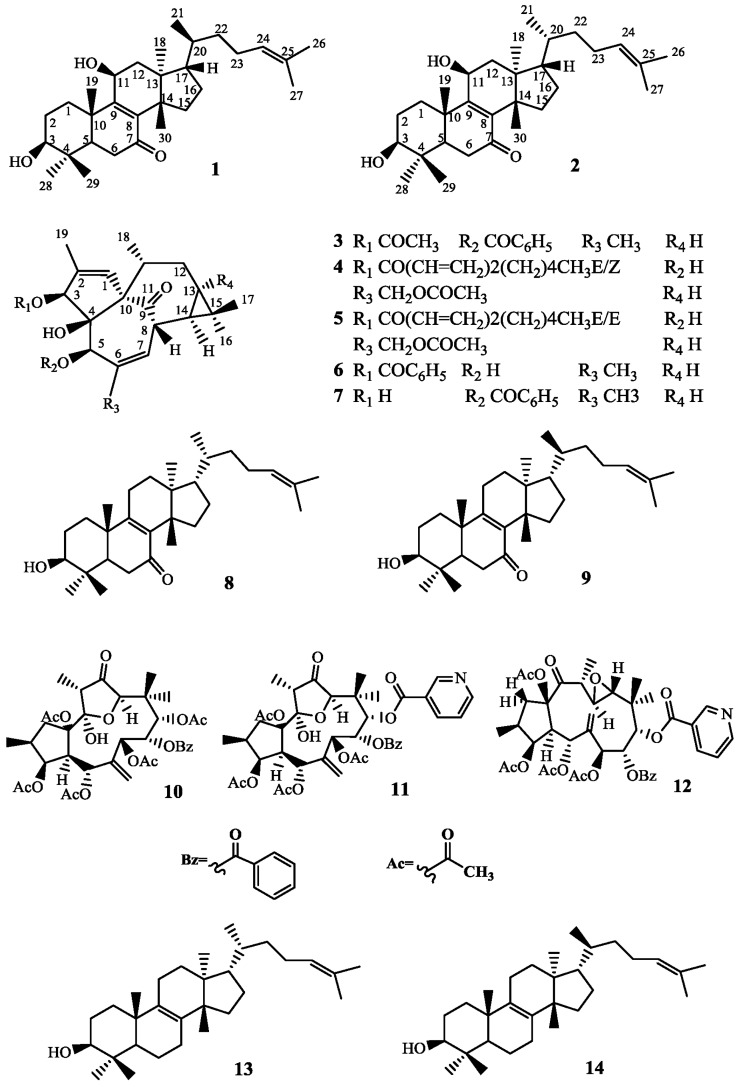
Chemical structures of Compounds **1**–**14**.

The plants of *Euphorbia* contain more than 2000 species spread all over the world, and about 80 species distribute in China [1,2]. The dried root of *Euphorbia kansui* has long been used for the treatment of asthma, edema and ascites in traditional Chinese medicine. The structure type of the compounds in *Euphorbia kansui* are diterpenes, triterpenes, flavonoids, phenolic and acids [3,4,5]. Among them, diterpenes and triterpenoids are the main compounds in *Euphorbia kansui*, which show a wide range of pharmacological activities, such as antiviral, skin irritating and modulation of multidrug resistance effects [6,7,8,9]. A new tirucallane-type triterpene named tirucalla-8,24-diene-3β,11β-diol-7-one (**1**) was first isolated from natural plants, and an euphane-type triterpene named eupha-8,24-diene-3β,11β-diol-7-one (**2**) (Figure 1) was isolated from *Euphorbia kansui* for the first time in our present study. The two compounds were identified by 1D and 2D NMR including Heteronuclear Single Quantum Coherence (HSQC), Heteronuclear Multiple-Bond Correlation (HMBC), COrrelation SpectroscopY (COSY), Nuclear Overhauser Effect Spectroscopy (NOESY) and HR-ESI-MS data.

## 2. Results and Discussion

**Figure 2:**
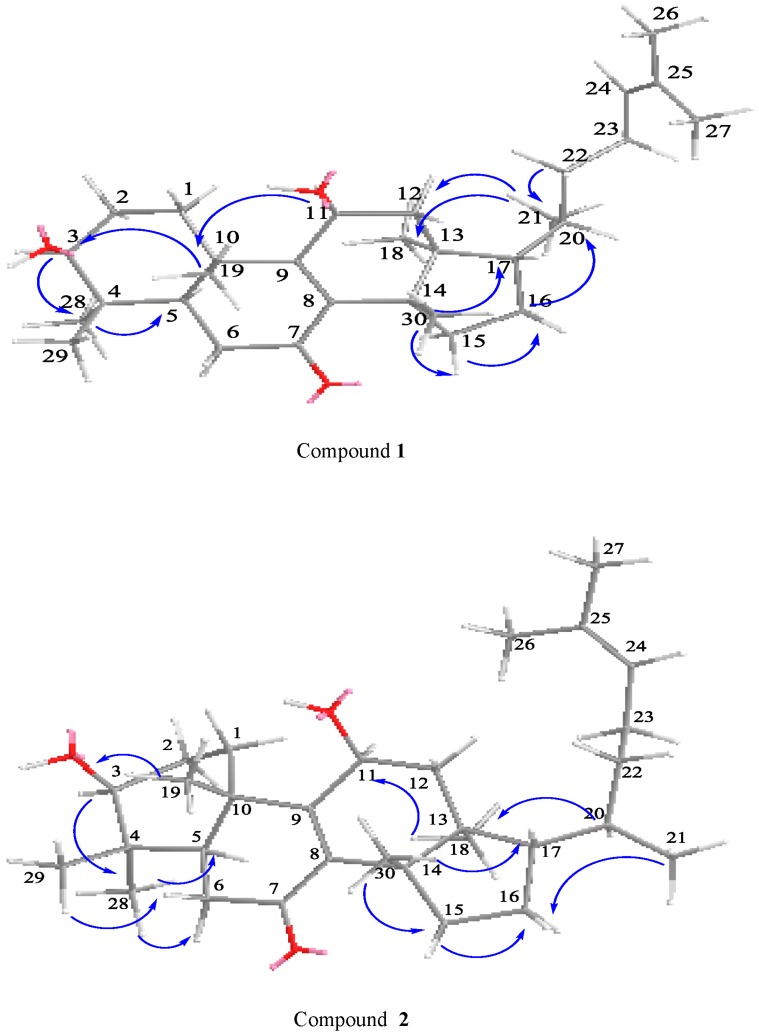
Key NOESY correlations for **1** and **2**.

**Figure 3:**
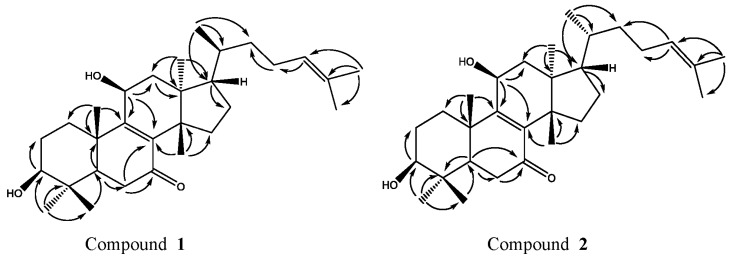
Key HMBC correlations for **1** and **2**.

Compound **1** was obtained as a white powder. The molecular formula C_30_H_48_O_3_ was established by HR-ESI-MS (*m*/*z* 457.3770 [M + H]^+^, calcd. 457.3682) (Figure 1-1), IR (KBr) *ν*_max_ 3371, 2977, 2861, 1635, 1456, 1376, 1036, 622 cm^−1^ (Figure 1-2), UV (MeOH) *λ*_max_ 253 nm (Figure 1-3) (Figures 1-1, 1-2, 1-3, see the Supplementary Materials); The comparison of ^1^H-NMR, ^13^C-NMR (Table 1 and Table 2, Figures 1-4 and 1-5, see the Supplementary Materials) and NOESY data (Figure 2) showed that compound **1** and the known compound **2** [10] were semblable in structure, except that a Hydrogen at C-11 of compound **2** was superseded by a hydroxyl group in compound **1**, which was verified by correlations of H-11 (δ_H_ 4.70, t, *J* = 8.2 Hz) with C-8 (δ_C_ 140.46), C-9 (δ_C_ 161.25) and C-12 (δ_C_ 42.79) in HMBC spectrum (Figure 3). Compared with ^1^H-NMR and ^13^C-NMR of **1** and **2**, we can clearly see the differences between compound **2**: H-C(21) δ_H_ 0.88, H-C(22) δ_H_ 1.08–1.13 (m), 1.56–1.62 (m), C-20 δ_C_ 35.61, C-22 δ_C_ 35.51) and compound **1**: H-C(21) δ_H_ 0.94, H-C(22) δ_H_ 1.03–1.12 (m), C-20 δ_C_ 36.03, C-22 δ_C_ 36.24. In the HSQC plot (Figure 1-6, see the Supplementary Materials), δ_H_ 1.03–1.12 and 1.40–1.47 showed correlations with C(22), C(20) at δ_C_ 36.24, 36.03 respectively, which indicated compound **1**, H-C(22) δ_H_ 1.03–1.12 (m). The relative configuration of **1** was determined by the ^1^H-NMR (Table 1) and NOESY data. ^1^H-NMR chemical shift of CH_3_-21 (δ_H_ 0.94, d, *J* = 6.6 Hz) confirmed that compound **1** was classified as the tirucallane series [11,12,13]. The large coupling constants H-C(3) (δ_H_ 3.31, *J* = 9.5, 6.4 Hz) obviously indicated that the 3-OH group was in equatorial β-position [10,14]. The NOESY correlations (Figure 2) H-C(3) (δ_H_ 3.31)/H-C(5) (δ_H_ 1.65–1.68), H-C(3) (δ_H_ 3.31)/CH_3_-28 (δ_H_ 1.00), H-C(11) (δ_H_ 4.70)/CH_3_-18 (δ_H_ 0.71), CH_3_-30 (δ_H_ 1.15)/H-C(17) (δ_H_ 1.54–1.61), CH_3_-29 (δ_H_ 0.92)/CH_3_-19 (δ_H_ 1.28), CH_3_-18 (δ_H_ 0.71)/CH_3_-19 (δ_H_ 1.28), showed that H-C(3), H-C(5), H-C(17), CH_3_-28, were all in *α*-orientation, whereas 11-OH, CH_3_-19, CH_3_-30 and CH_3_-29 were all in β-orientation. Furthermore, compound **1** showed NOESY correlations between CH_3_-18 and H-C(20) (δ_H_ 1.40–1.47) and CH_3_-21, between CH_3_-21 and H-12β (δ_H_ 2.38–2.46). These correlations were consistent with those of tirucallane-type triterpenes [14]. As a result, the structure of compound **1** was identified as tirucalla-8,24-diene-3β,11β-diol-7-one.

The comparison of ^1^H-NMR, ^13^C-NMR (Table 1 and Table 2) and NOESY data (Figure 2) showed that compound **2** and the known Compound **1** [10] were semblable in structure, except that a Hydrogen at C-11 of Compound **1** was superseded by a hydroxyl group in compound **2**, which was proven by correlations of H-11 (δ_H_ 4.69, t, *J* = 8.2 Hz) with C-8 (δ_C_ 140.44), C-9 (δ_C_ 161.30) and C-12 (δ_C_ 42.83) in HMBC spectrum (Figure 3). The large coupling constants H-C(3) (δ_H_ 3.31, *J* = 9.5, 6.4 Hz) obviously indicated that the 3-OH group was in equatorial β-position [11,12]. The NOESY correlations (Figure 2) H-C(3) (δ_H_ 3.31)/H-C(5) (δ_H_ 1.62–1.68), H-C(3) (δ_H_ 3.31)/CH_3_-28 (δ_H_ 1.01), H-C(11) (δ_H_ 4.69)/CH_3_-18 (δ_H_ 0.73), CH_3_-30 (δ_H_ 1.16)/H-C(17) (δ_H_ 1.55–1.60), CH_3_-29 (δ_H_ 0.93)/CH_3_-19 (δ_H_ 1.27), CH_3_-18 (δ_H_ 0.73)/CH_3_-19 (δ_H_ 1.27), showed that H-C(3), H-C(5), H-C(17), and CH_3_-28 were all in *α*-orientation, whereas 11-OH, CH_3_-19, CH_3_-30 and CH_3_-29 were in β-orientation, and no correlations between CH_3_-21/CH_3_-18 [10,15] indicated that it belonged to the euphane-type triterpenes. The ^1^H-NMR chemical shift of CH_3_-21 (δ_H_ 0.88, d, *J* = 6.4 Hz) further confirmed that compound **2** pertained to the euphane rather than the tirucallane series [12,13]. As a result, compound **2** was elucidated to be eupha-8,24-diene-3β,11β-diol-7-one.

All 12 of the known compounds (**3**–**14**) were identified according to the spectroscopic data (^1^H-NMR, ^13^C-NMR, see the Supplementary Materials) together with the comparsion of those reported, kansuiphorin C (**3**) [16], 3-*O*-(*2*′*E*,*4*′*Z*-decadienoyl)-20-*O*-acetylingenol (**4**) [4], 3-*O*-(*2*′*E*,*4*′*E*-decadienoyl)-20-*O*-acetylingenol (**5**) [4], 3-*O*-benzoyl-20-deoxyingenol (**6**) [4], 5-*O*-benzoyl-20-deoxyingenol (**7**) [4], kansenone (**8**) [10], *epi*-kansenone (**9**) [10], kansuinin A (**10**) [4,17], kansuinin D (**11**) [4], kansuinin E (**12**) [4], euphol (**13**) [4,18], and tirucallol (**14**) [4,19].

Compounds **1** and **2** were assessed for their inhibitory effects on HCT-116, MKN-45 and MCF-7 cell lines (Table 3), as well as L-O2 and GES-1 cell lines (Table 4). The results show that compounds **1** and **2** inhibit normal cells (L-O2 and GES-1) less than cancer cells (HCT-116, MKN-45 and MCF-7), and compounds **1** and **2** presented definite anticancer activities with IC_50_ values of 20.84 ± 1.28 and 33.97 ± 2.15 µΜ against HCT-116 cells, 10.18 ± 1.36 and 14.95 ± 1.82 µΜ against MKN-45 cells, and 10.82 ± 1.18 and 20.11 ± 2.16 µΜ against MCF-7 cells, respectively. kansenone induces apoptosis through both the death receptor and mitochondrial pathways [5], and compounds **1** and **2** were similar with kansenone in structure, excluding that a Hydrogen at C-11 of kansenone was superseded by a hydroxyl group in compounds **1** and **2**, thus compounds **1** and **2** may induce apoptosis in the same way as kansenone. Further research will be conducted on the anticancer mechanism of compounds **1** and **2**.

## 3. Materials and Methods

### 3.1. General Experimental Procedures

HPLC: Hanbon NP 7000 (Jiangsu Hanbang Technology Companies, Huaian, China) Serials pump with an NU 3000 Serials UV-Vis detector (Jiangsu Hanbang Technology Companies, Huaian, China), Phecda Si (20 × 250 mm, 5 μm); Waters 1525 with a 2996 Diode Array Detector (DAD) (Waters, Milford, CT, USA), XBrige-Prep C_18_ (150 × 19 mm, 5 μm), (Ultimate XB-C8, 30 × 150 mm, 5 μm). IR spectra were gained on a Nicole Is5 of Thermo Fisher spectrophotometer (Nicolet Instrument Corporation, Madison, WI, USA). The NMR spectra were measured on Avance 400 spectrometers (Bruker, Karlsruhe, Germany), with TMS as an internal standard. The UV spectra were measured on a Shimadzu UV-2401 UV-Vis spectrophotometer (Shimadzu, Kyoto, Japan). The HR-ESI-MS data were obtained by using a LTQ Orbitrap MS (Thermo Fisher Scientific, San Jose, CA, USA). Column chromatography (CC): silica gel (200–300 mesh, Qingdao Marine Chemical Industry, Qingdao, China).

### 3.2. Plant Materials

The dried root of *Euphorbia kansui* was collected from Red River valley of Baoji, Shaanxi Province of China, in October 2015 and was identified by Prof. Yu Ping Tang (college of pharmacy, Nanjing University of Chinese Medicine, Nanjing, China). The voucher specimen (20151020) has been deposited in the Herbarium of college of pharmacy, Nanjing University of Chinese Medicine (Nanjing, China).

### 3.3. Extraction and Isolation

The dried roots of *Euphorbia kansui* (12.2 kg) were extracted twice (each time for 2 h) with 95% EtOH under reflux to give the 95% EtOH extract (871.9 g) by evaporation of the solvent under reduced pressure, and then the 95% EtOH extract was extracted with ethyl acetate (EtOAc) to obtain ethyl acetate extract (530.8 g). Finally, the fraction of EtOAc was subjected to silica gel column chromatography (14 × 59 cm) with a gradient elution (Pet and ethyl acetate, 100:1–1:1) to get fractions A-T. Fr. G (2 g) was eluted with Pet:EtOAc (100:20). Compound **1** (40.8 mg) was isolated by HPLC (Pet:EtOAc, 100:38), and further purified by reversed-phase HPLC (MeCN:H_2_O, 70:30) flow rate 16 mL/min (t_R_ 15.342 min). Compound **2** (70.3 mg) was isolated by HPLC (Pet:EtOAc, 100:30), and further purified by reversed-phase HPLC (MeCN:H_2_O, 70:30) flow rate 16 mL/min (t_R_ 16.060 min). Fr. A (18 g) was eluted with Pet:EtOAc (100:3). Compound **13** (6.082 g) was isolated by HPLC (MeCN:H_2_O, 95:5) with (Ultimate XB-C8, 30 × 150 mm, 5 μm) flow rate 16 mL/min (t_R_ 35.452 min). Compound **14** (782.5 mg) was isolated by HPLC (MeCN:H_2_O, 95:5) with (Ultimate XB-C8, 30 × 150 mm, 5 μm) flow rate 16 mL/min (t_R_ 33.645 min). Fr. B (3 g) was eluted with Pet:EtOAc (100:5). Compound **6** (320 mg) was isolated by HPLC (Pet:EtOAc, 100:10) flow rate 16 mL/min (t_R_ 24.003 min). Compound **7** (46 mg) was isolated by HPLC (Pet:EtOAc, 100:10) flow rate 16 mL/min (t_R_ 22.209 min). Fr. C (5 g) was eluted with Pet:EtOAc (100:6). Compound **3** (920 mg) was isolated by HPLC (Pet:EtOAc, 100:11) flow rate 16 mL/min (t_R_ 24.128 min). Fr. D (2 g) was eluted with Pet:EtOAc (100:8). Compound **8** (161 mg) was isolated by HPLC (Pet:EtOAc, 100:15) flow rate 16 mL/min (t_R_ 23.218 min). Compound **9** (28 mg) was isolated by HPLC (Pet:EtOAc, 100:15) flow rate 16 mL/min (t_R_ 20.674 min). Fr. E (2 g) was eluted with Pet:EtOAc (100:13). Compound **4** (80 mg) was isolated by HPLC (Pet:EtOAc, 100:20) flow rate 16 mL/min (t_R_ 21.097 min). Compound **5** (40 mg) was isolated by HPLC (Pet:EtOAc, 100:20) flow rate 16 mL/min (t_R_ 24.298 min). Fr. H (10 g) was eluted with Pet:EtOAc (100:55). Compound **10** (186 mg) was isolated by HPLC (Pet:EtOAc, 100:40), and further purified by reversed-phase HPLC (MeCN:H_2_O, 70:30) flow rate 16 mL/min (t_R_ 10.502 min). Compound **11** (60 mg) was isolated by HPLC (Pet:EtOAc, 100:40), and further purified by reversed-phase HPLC (MeCN:H_2_O, 70:30) flow rate 16 mL/min (t_R_ 9.702 min). Compound **12** (80 mg) was isolated by HPLC (Pet:EtOAc, 100:40), and further purified by reversed-phase HPLC (MeCN:H_2_O, 70:30) flow rate 16 mL/min (t_R_ 11.302 min).

### 3.4. Cytotoxicity Test

Cytotoxicity of two compounds against HCT-116, MKN-45 and MCF-7 cancer cell lines (American Type Culture Collection, ATCC, Manassas, VA, USA), normal liver cell L-O2 (Zhongqiaoxinzhou Biotech, Shanghai, China) and gastric epithelial cell GES-1 (Nanjingkebai Biotech, Nanjing, China) were evaluated with the MTT (3-(4,5-dimethylthiazol-2-yl)-2,5-diphenylt-etrazolium bromide) method as described in the literature [20,21]. All cells were cultured in Dulbecco’s modified Eagle’s medium (DMEM) supplemented with 10% fetal bovine serum, three cancer cells were seeded in 96-well plate at a concentration of 8 × 10^4^ cells/mL, two normal cells L-O2 and GES-1 were seeded in 96-well plate at a concentration of 1 × 10^4^ cells/mL and 1 × 10^5^ cells/mL respectively [21], and incubated for 24 h in humidifyed atmosphere with 5% CO_2_ at 37 °C. One hundred microliters of cells were treated with compound **1** and **2** at different doses of 1.25, 2.5, 5, 10, 20 and 40 μg/mL in dimethyl sulfoxide (DMSO) in triplicate for 48 h at 37 °C with 5% CO_2_. Then, each of them were added with 20 μL of MTT (5.0 mg/mL) and incubated for further 4 h, the growth medium was removed from all the wells. Finally, 150 μL DMSO were added to every sample. Cisplatin (Qilu pharmaceutical, Jinan, China) and 5-fluorouracil (5-Fu) (Sichuan Kangyi, pharmaceutical, Chengdu, China) served as positive control. Absorbance was determined by a microplate spectrophotometer at 490 nm.

## 4. Conclusions

The article reported two compounds, **1** and **2**, which are triterpenes, as well as twelve other known compounds (**3**–**14**). Compound **1** is a new tirucallane-type triterpene named tirucalla-8,24-diene-3β,11β-diol-7-one. Compound **2** was isolated from *Euphorbia kansui* for the first timeand named eupha-8,24-diene-3β,11β-diol-7-one. They also display effective anticancer activities against HCT-116, MKN-45 and MCF-7 cells. As we all know, *Euphorbia kansui* has pharmacological activities including tumor inhibition and antiviral effects [22,23]; this study further confirmed kansui may be a potential candidate for anticancer including colon cancer, gastric cancer and breast cancer and inferred that compounds **1** and **2** may be the main material basis of anticancer for *Euphorbia kansui*.

Some features about compounds **1**, **2**, **8**, **9**, **13** and **14** may be drawn based on their chemical structures. Above all, the CH_3_-21 of compounds **9** and **14** were all in β-orientation and their ^1^H-NMR chemical shift of CH_3_-21 at (δ_H_ 0.94, d, *J* = 6.6 Hz), whereas the CH_3_-21 of compounds **8** and **13** were all in *α*-orientation and their ^1^H-NMR chemical shift of CH_3_-21 at (δ_H_ 0.88, d, *J* = 6.4 Hz) [4,18,19]. The C-20 and C-22 of compound **9** had a chemical shift greater than compound **8**. The C-20 and C-22 of compound **14** was larger than compound **13** (Table 5), which were identical with the ^1^H-NMR and ^13^C-NMR of compounds **1** and **2**, respectively. Then, the polarity order of compound **9** was larger than compound **8**. Compound **14** is also greater than **13** in the same way, which were also the same as the polarity order of compounds **1** and **2**. Thus, the different positions of CH_3_-21 of compounds **1** and **2**, **8** and **9**, and **13** and **14** may have a good correlation with their polarity order.

## Figures and Tables

**Table 1:** ^1^H-NMR data for compounds **1** and **2**.

Position	Compound 1	Compound 2
1*α*	1.56–1.62 (m)	1.52–1.60 (m)
1β	2.41–2.47 (m)	2.40–2.46 (m)
2	1.70–1.79 (m)	1.70–1.79 (m)
3*α*	3.31 (dd, *J* = 6.4, 9.6)	3.31 (dd, *J* = 6.4, 9.6)
5	1.65–1.68 (m)	1.62–1.68 (m)
6	2.40–2.47 (m)	2.40–2.48 (m)
11	4.70 (t, *J* = 8.2)	4.69 (t, *J* = 8.2)
12*α*	1.76–1.81 (m)	1.74–1.81 (m)
12β	2.38–2.46 (m)	2.35–2.43 (m)
15*α*	2.10–2.12 (m)	2.12–2.19 (m)
15β	1.42–1.46 (m)	1.40–1.46 (m)
16*α*	1.93–1.99 (m)	1.90–1.94 (m)
16β	1.28–1.33 (m)	1.28–1.32 (m)
17	1.54–1.61 (m)	1.55–1.60 (m)
18	0.71 (s)	0.73 (s)
19	1.28 (s)	1.27 (s)
20	1.40–1.47 (m)	1.40–1.46 (m)
21	0.94 (d, *J* = 6.6)	0.88 (d, *J* = 6.4)
22	1.03–1.12 (m)	1.08–1.13 (m) 1.56–1.62 (m)
23	1.83–1.91 (m) 2.01–2.08 (m)	1.84–1.90 (m) 1.98–2.02 (m)
24	5.09(t, *J* = 7.2)	5.09 (t, *J* = 7.2)
26	1.70 (s)	1.70 (s)
27	1.62 (s)	1.63 (s)
28	1.00 (s)	1.01 (s)
29	0.92 (s)	0.93 (s)
30	1.15 (s)	1.16 (s)

Record in CDCl_3_, 400 MHz for ^1^H, δ in ppm, *J* = Hz.

**Table 2:** ^13^C-NMR data of compounds **1** and **2**.

Position	Compound 1	Compound 2
1	33.68	33.67
2	27.37	27.36
3	78.27	78.26
4	39.07	39.06
5	49.25	49.25
6	35.84	35.84
7	200.10	200.16
8	140.46	140.44
9	161.25	161.30
10	39.58	39.58
11	68.11	68.11
12	42.79	42.83
13	46.19	46.19
14	47.95	48.02
15	31.88	31.81
16	27.77	27.80
17	49.13	48.69
18	16.06	16.24
19	19.70	19.67
20	36.03	35.61
21	18.68	18.76
22	36.24	35.51
23	24.86	24.82
24	124.98	124.90
25	131.09	131.14
26	25.68	25.73
27	17.62	17.70
28	27.59	27.59
29	15.18	15.19
30	25.62	25.70

Record in CDCl_3_, 100 MHz for ^13^C, δ in ppm, *J* = Hz.

**Table 3:** Cytotoxicity of compounds **1** and **2** against three human cancer cell lines.

Compound	IC_50_ (μΜ)		
	HCT-116	MKN-45	MCF-7
**1**	20.84 ± 1.28	10.18 ± 1.36	10.82 ± 1.18
**2**	33.97 ± 2.15	14.95 ± 1.82	20.11 ± 2.16
Cisplatin	8.465 ± 0.84	6.142 ± 1.12	9.035 ± 0.92
5-Fu	6.172 ± 2.03	2.624 ± 2.06	1.629 ± 1.42

**Table 4:** Cytotoxicity of compounds **1** and **2** against two human normal cell lines.

Compound	IC_50_ (μΜ)	
	L-O2	GES-1
**1**	56.98 ± 1.74	40.99 ± 0.85
**2**	49.89 ± 2.12	40.27 ± 1.28

**Table 5:** ^13^C-NMR data of compounds **8**, **9**, **13** and **14**.

Position	Compound 8	Compound 9	Compound 13	Compound 14
1	34.61	34.61	35.26	35.26
2	27.40	27.41	27.95	27.92
3	78.07	78.06	79.00	79.03
4	38.82	38.83	38.94	38.94
5	48.19	48.24	50.97	50.97
6	35.77	35.78	18.95	18.95
7	198.37	198.35	27.68	27.67
8	138.94	138.93	134.03	134.08
9	165.46	165.48	133.55	133.51
10	39.27	39.27	37.24	37.27
11	23.73	23.67	21.53	21.45
12	29.95	29.87	30.90	30.80
13	44.62	44.61	44.12	44.11
14	47.68	47.61	50.03	50.12
15	31.39	31.45	29.77	28.83
16	28.67	28.65	28.14	28.05
17	48.24	48.76	49.64	49.96
18	15.73	15.54	15.63	15.52
19	18.60	18.61	20.15	20.14
20	35.65	36.16	35.88	36.33
21	18.90	18.75	18.92	18.69
22	35.52	36.35	35.43	36.40
23	24.74	24.91	24.77	24.94
24	125.07	125.12	125.22	125.26
25	131.04	131.01	130.08	130.90
26	25.76	25.73	17.69	17.62
27	17.71	17.65	25.74	25.71
28	27.28	27.29	27.92	27.92
29	15.07	15.07	15.53	15.43
30	24.42	24.31	24.47	24.36

Record in CDCl_3_, 100 MHz for ^13^C, δ in ppm, *J* = Hz.

## Supplementary Materials

Supplementary materials are available online.

## References

[1] Geng T., Huang H.Y., Ding A.W., Zhang L. (2008). Irritation and diarrhea effect of different polar parts of *Euphorbia kansui* T. and vinegar-preparing *Euphorbia kansui* T.. Cent. South Pharm..

[2] Wang L., Ma Y.T., Sun Q.Y., Li X.N., Yan Y., Yang J., Yang F.-M., Liu F.-Y., Zang Z., Wu X.H. (2015). Structurally diversified diterpenoids from *Euphorbia dracunculoides*. Tetrahedron.

[3] Wang H., Wang J., Luo J., Kong L. (2013). Isolation of ingenol-type diterpenoids from *Euphorbia kansui* by offline coupling of HPLC-ESI-MSn and HSCCC. Sep. Sci. Technol..

[4] Wang L.Y. (2003). The Bioactive Study of Constituents in Kansui. Ph.D. Thesis.

[5] Cheng F., Yang Y., Zhang L., Cao Y., Yao W., Tang Y., Ding A. (2015). A natural triterpene derivative from *Euphorbia kansui* inhibits cell proliferation and induces apoptosis against rat intestinal epithelioid cell line in vitro. Int. J. Mol. Sci..

[6] Shi Q.W., Su X.H., Kiyota H. (2008). Chemical and pharmacological research of the plants in genus *Euphorbia*. Chem. Rev..

[7] Zheng W.F., Cui Z., Zhu Q. (1998). Cytotoxicity and antiviral activity of the compounds from *Euphorbia kansui*. Planta Med..

[8] Li X.R., Zhang Y.D., Tang H.H. (2002). Study of auxiliary therapeutic effect of kansui root on patients with severe acute pancreatitis. China J. Mod. Med..

[9] Yan X., Zhang L., Guo J., Cao Y., Shang E., Tang Y., Ding A., Duan J.A. (2014). Processing of kansui roots stir-baked with vinegar reduces kansui-induced hepatocyte cytotoxicity by decreasing the contents of toxic terpenoids and regulating the cell apoptosis pathway. Molecules.

[10] Wang L.Y., Wang N.L., Yao X.S., Miyata S., Kitanaka S. (2003). Euphane and tirucallane triterpenes from the roots of *Euphorbia kansui* and their in vitro effects on the cell division of Xenopus. J. Nat. Prod..

[11] Ahmed E., Malik A., Ferheen S., Afza N., Lodhi M.A., Choudhary M.I. (2006). Chymotrypsin inhibitory triterpenoids from *Silybum marianum*. Chem. Pharm. Bull..

[12] Abe I., Rohmer M. (1994). Enzymic cyclization of 2,3-dihydrosqualene and squalene 2,3-epoxide by squalene cyclases: From pentacyclic to tetracyclic triterpenes. J. Chem. Soc. Perkin Trans. 1.

[13] Mishra M., Shukla Y.N., Kumar S. (2000). *Euphane triterpenoid* and lipid constituents from *Butea monosperma*. Phytochemistry.

[14] Wang S., Liang H., Zhao Y., Wang G., Yao H., Kasimu R., Wu Z., Li Y., Huang J., Wang J. (2016). New triterpenoids from the latex of *Euphorbia resinifera* Berg. Fitoterapia.

[15] Akihisa T., Yasukawa K., Kimura Y., Takase S.I., Yamanouchi S., Tamura T. (1997). Triterpene alcohols from camellia and sasanqua oils and their anti-inflammatory effects. Chem. Pharm. Bull..

[16] Pan D.-J., Hu C.-Q., Chang J.-J., Lee T.T.-Y., Chen Y.-P., Hsu H.-Y., Mcphail D.R., Mcphail A.T., Lee K.-H. (1991). Kansuiphorin-C and-D, cytotoxic diterpenes from *Euphorbia kansui*. Phytochemistry.

[17] Uemura D., Hirata Y., Chen Y.P., Hsu H.Y. (1975). The structure of kansuinine A, a new multi-oxygenated diterpene. Tetrahedron Lett..

[18] Gewali M.B., Hattori M., Tezuka Y., Kikuchi T., Namba T. (1990). Constituents of the latex of *Euphorbia antiquorum*. Phytochemistry.

[19] Itoh T., Tamura T., Matsumoto T. (1976). Tirucalla-7, 24-dienol: A new triterpene alcohol from tea seed oil. Lipids.

[20] Gao J., Gao L., Zhang L., Yao W., Cao Y., Bao B., Ding A. (2015). 3-*O*-(2′*E*,4′*Z*-decadienoyl)-20-*O*-acetylingenol induces apoptosis in intestinal epithelial cells of rats via mitochondrial pathway. J. Ethnopharmacol..

[21] Zhang L., Gao L., Li Z., Yan X., Yang Y., Tang Y., Cao Y., Ding A. (2012). Bio-guided isolation of the cytotoxic terpenoids from the roots of *Euphorbia kansui* against human normal cell lines L-O2 and GES-1. Int. J. Mol. Sci..

[22] Shen J., Wang J., Shang E.X., Tang Y.P., Kai J., Cao Y.J., Zhou G.S., Tao W.W., Kang A., Su S.L. (2016). The dosage-toxicity-efficacy relationship of kansui and licorice in malignant pleural effusion rats based on factor analysis. J. Ethnopharmacol..

[23] Yan X., Zhang L., Cao Y., Yao W., Tang Y., Ding A. (2016). An Ingenol Derived from *Euphorbia kansui* Induces Hepatocyte Cytotoxicity by Triggering G0/G1 Cell Cycle Arrest and Regulating the Mitochondrial Apoptosis Pathway in Vitro. Molecules.

