# Supplementary material for: Chemical Constituents from Euphorbia kansui
Source: Molecules. 2017 Dec 8;22(12):2176. doi: 10.3390/molecules22122176 (PMC6150036; doi:10.3390/molecules22122176)
Supplement: Supplementary file 1 [file molecules-22-02176-s001.pdf]

Chemical Constituents from *Euphorbia kansui*

Qiao Zhang [1], Qin-Rong Zhou [1], Jian-Wei Lou [1], Pei-Dong Chen [1], Wei-Feng Yao [1], Wei-Wei Tao [1], Yu-Ping Tang [2], Guan-Cheng Dai [1], Kun Wang [1] and Li Zhang [1,*]

[1] Jiangsu Key Laboratory for High Technology Research of TCM Formulae, National and Local Collaborative Engineering Center of Chinese Medicinal Resources Industrialization and Formulae Innovative Medicine and Jiangsu Collaborative Innovation Center of Chinese Medicinal Resources Industrialization, Nanjing University of Chinese Medicine, Nanjing 210023, China; 18700081184@163.com (Q.Z.); r790507807@gmail.com (Q.-R.Z.); trustlou@163.com (J.-W.L.); chenpeidong1970@163.com (P.-D.C.); yaowf@njucm.edu.cn (W.-F.Y.); tw845@163.com (W.-W.T.); 18260028211@163.com (G.-C.D.); 18260028237@163.com (K.W.)

[2] College of Pharmacy and Shaanxi Collaborative Innovation Center of Chinese Medicinal Resources Industrialization, Shaanxi University of Chinese Medicine, Xi'an 712046, China; 2051001@sntcm.edu.cn

* Correspondence: zhangli@njucm.edu.cn; Tel.: +86-138-5147-2740; Fax: +86-025-8581-1524

Compound 1

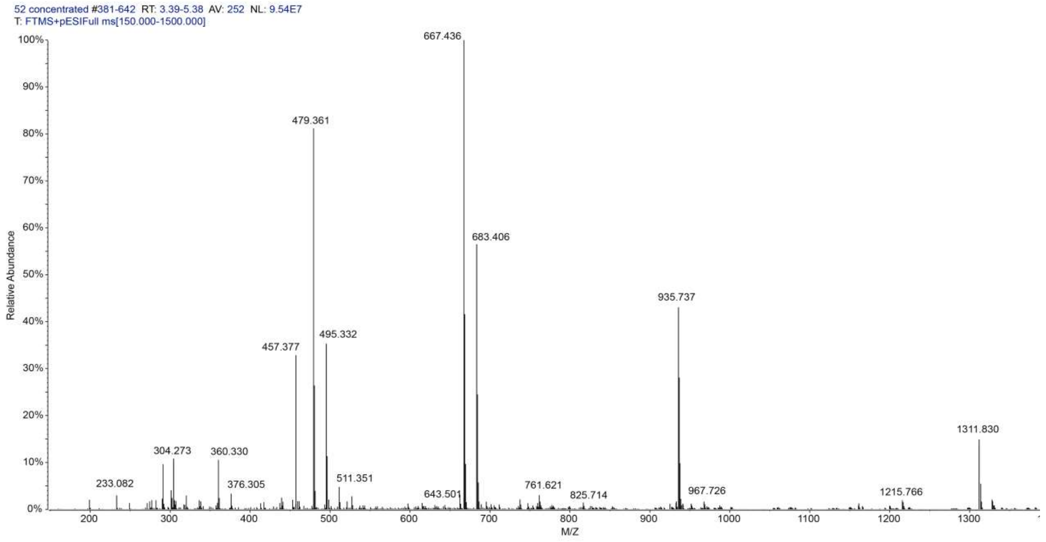

Figure 1-1. The HR-ESI-MS of tirucalla-8,23-diene-3*β*,11*β*-diol-7-one

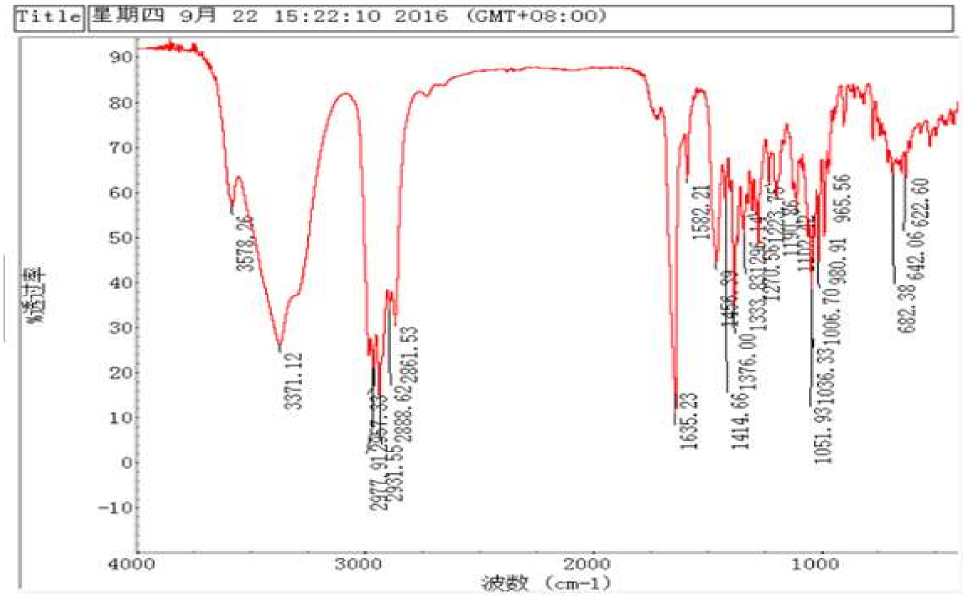

Figure 1-2. The IR of tirucalla-8,23-diene-3β,11β-diol-7-one

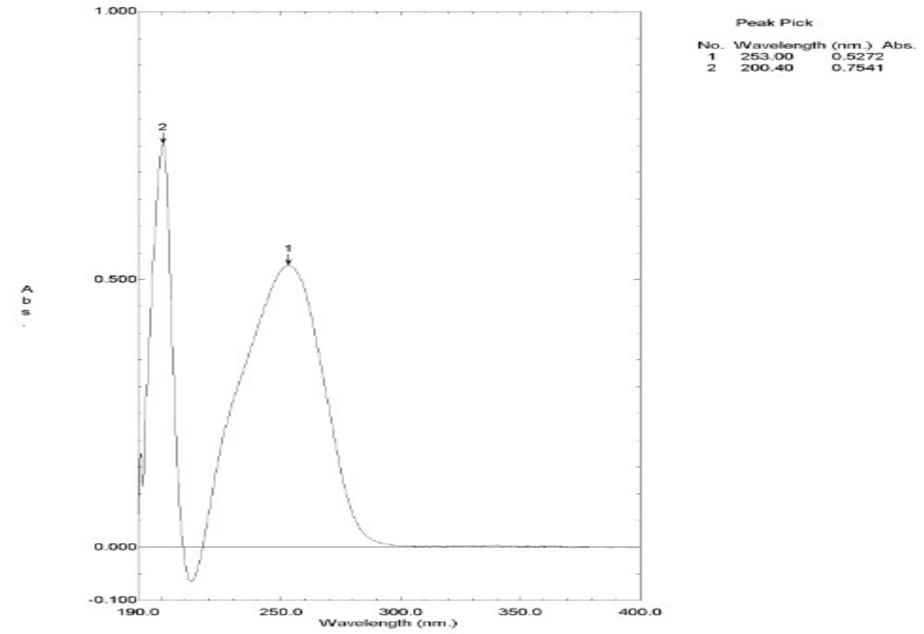

Figure 1-3. The UV of tirucalla-8,23-diene-3β,11β-diol-7-one

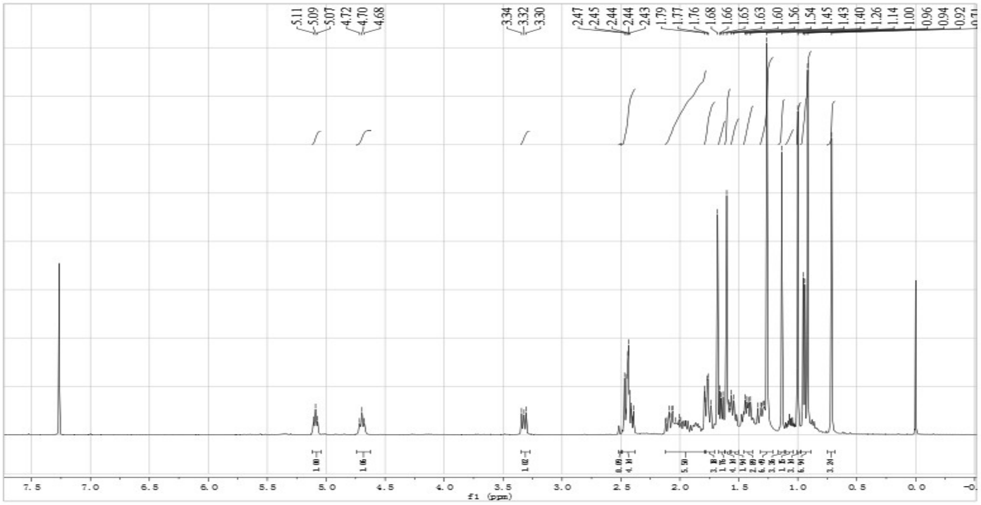

Figure 1-4. The ^{1}H-NMR of tirucalla-8,23-diene-3β,11β-diol-7-one

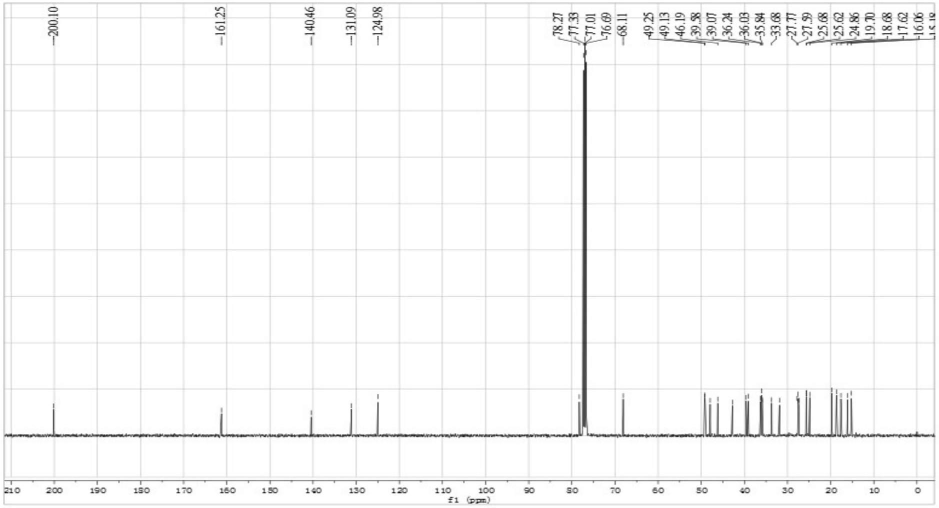

Figure 1-5. The ^{13}C-NMR of tirucalla-8,23-diene-3β,11β-diol-7-one

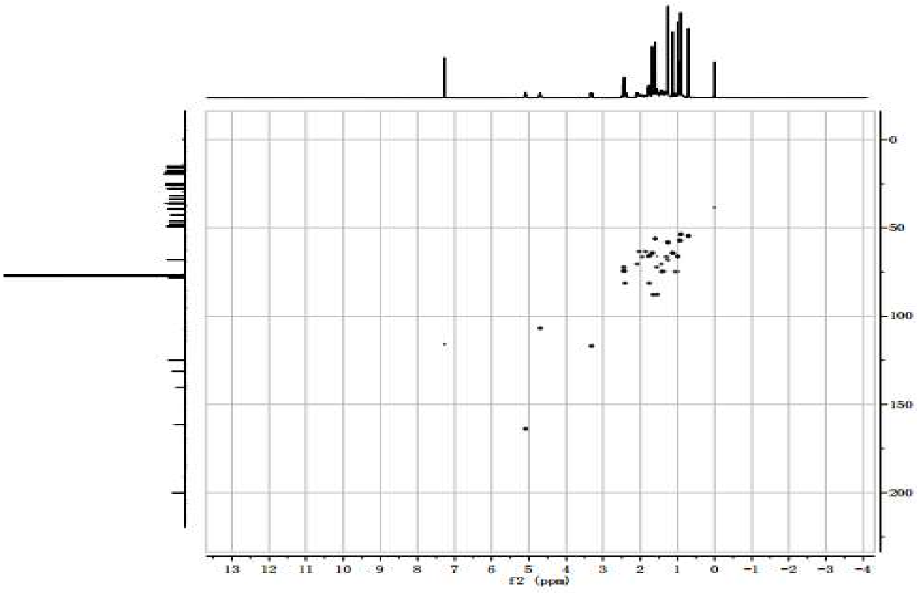

Figure 1-6. The 2D-HSQC of tirucalla-8,23-diene-3β,11β-diol-7-one

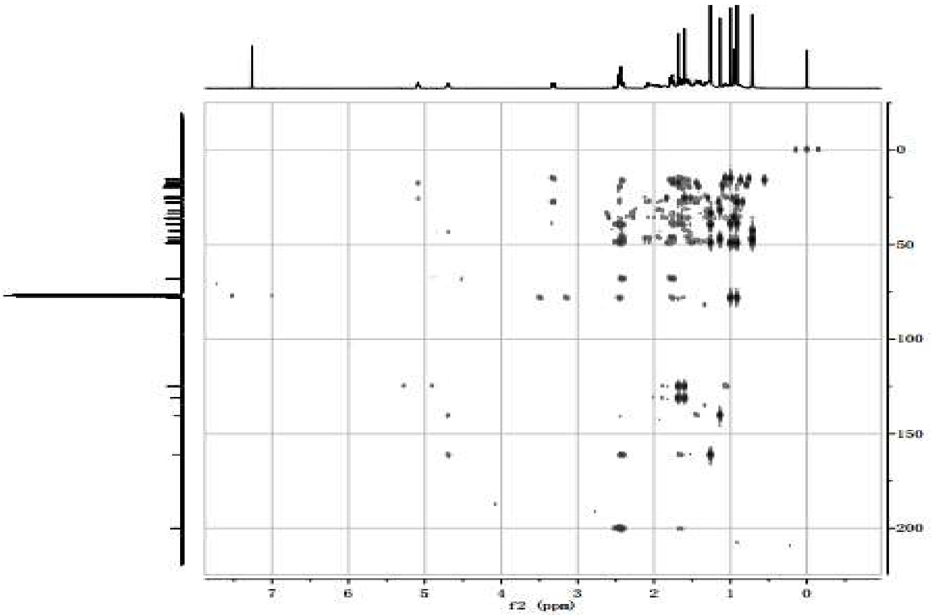

Figure 1-7. The 2D-HMBC of tirucalla-8,23-diene-3β,11β-diol-7-one

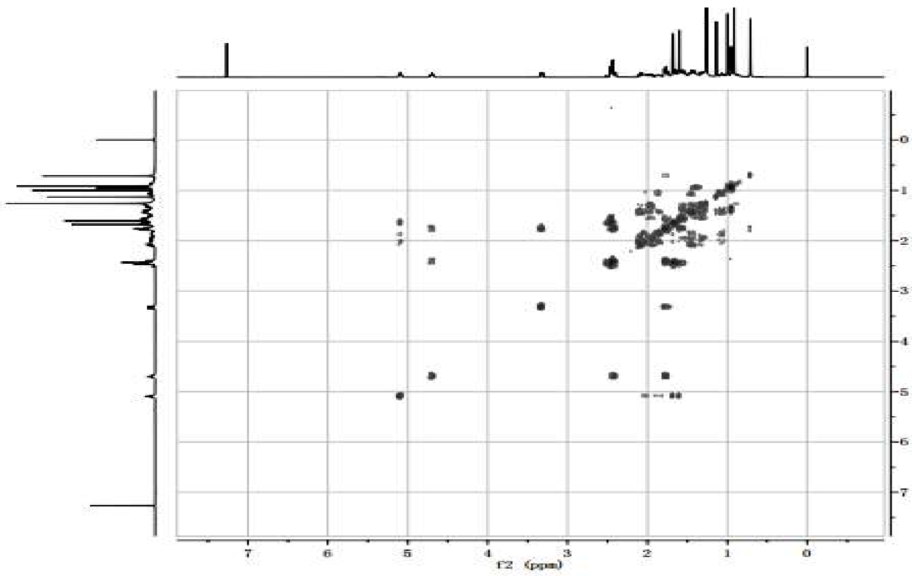

Figure 1-8. The 2D-COSY of tirucalla-8,23-diene-3β,11β-diol-7-one

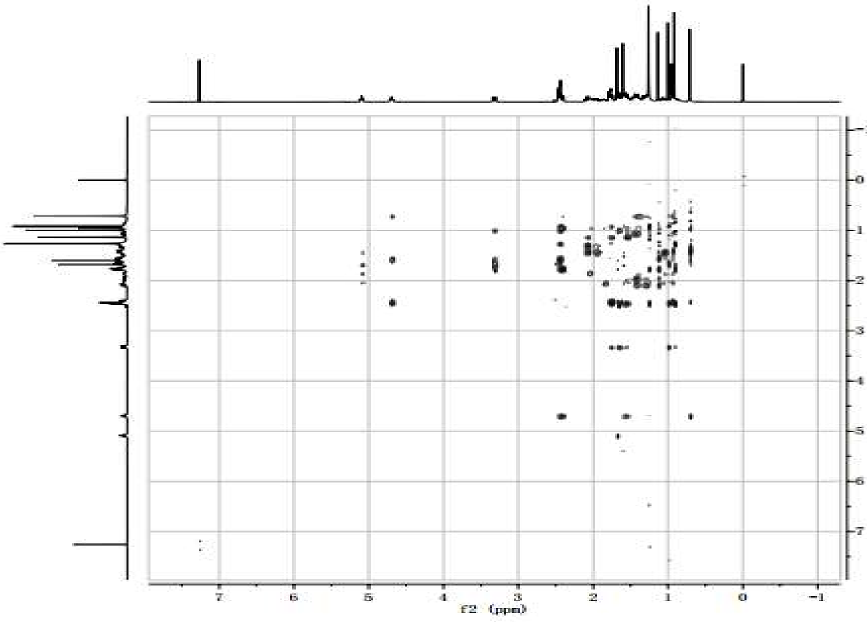

Figure 1-9. The 2D-NOESY of tirucalla-8,23-diene-3β,11β-diol-7-one

Compound 2

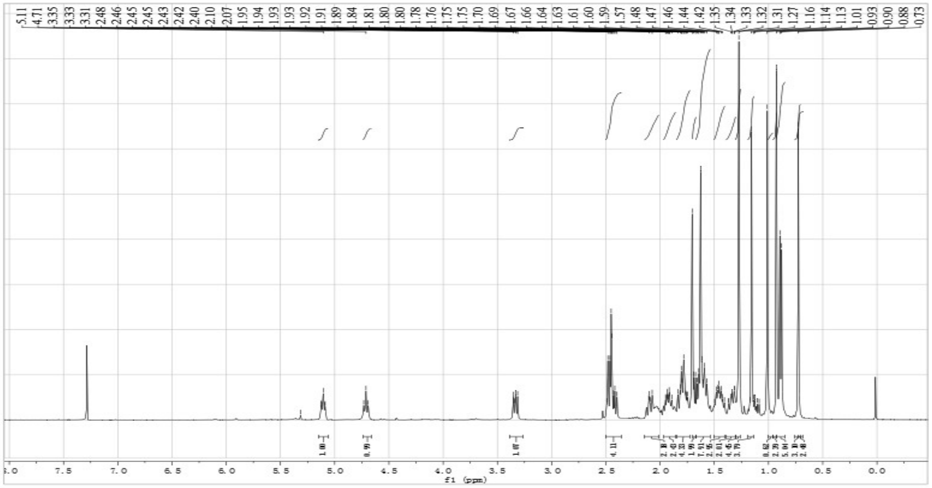

Figure 2-1. The ^{1}H-NMR of eupha-8,23-diene-3β,11β-diol-7-one

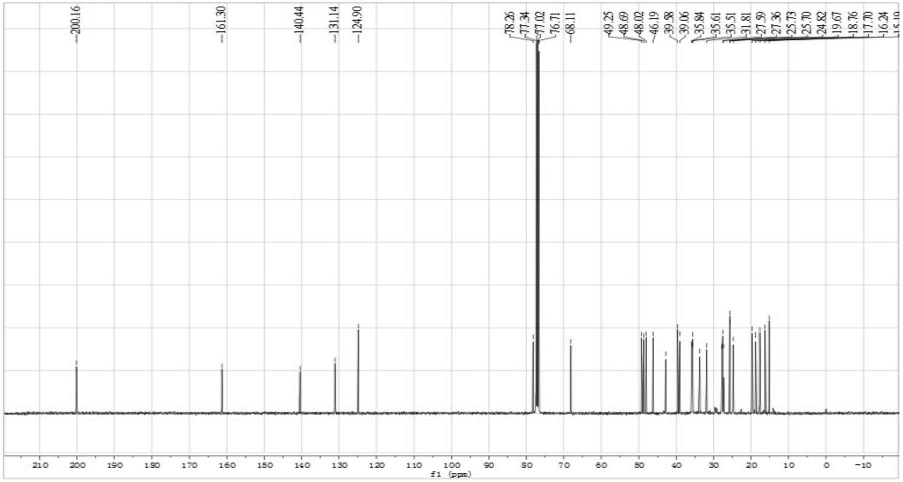

Figure 2-2. The ^{13}C-NMR of eupha-8,23-diene-3β,11β-diol-7-one

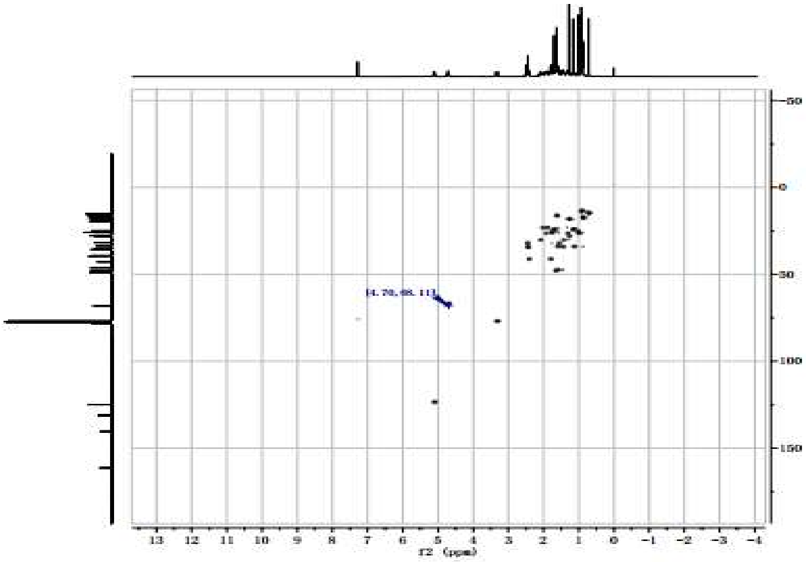

Figure 2-3. The 2D-HSQC of eupha-8,23-diene-3β,11β-diol-7-one

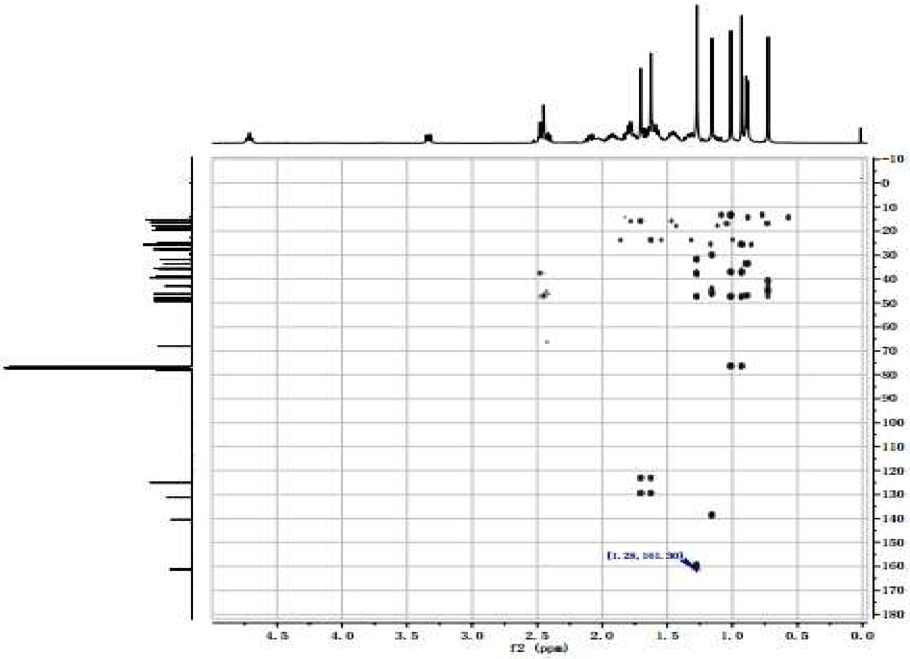

Figure 2-4. The 2D-HMBC of eupha-8,23-diene-3β,11β-diol-7-one

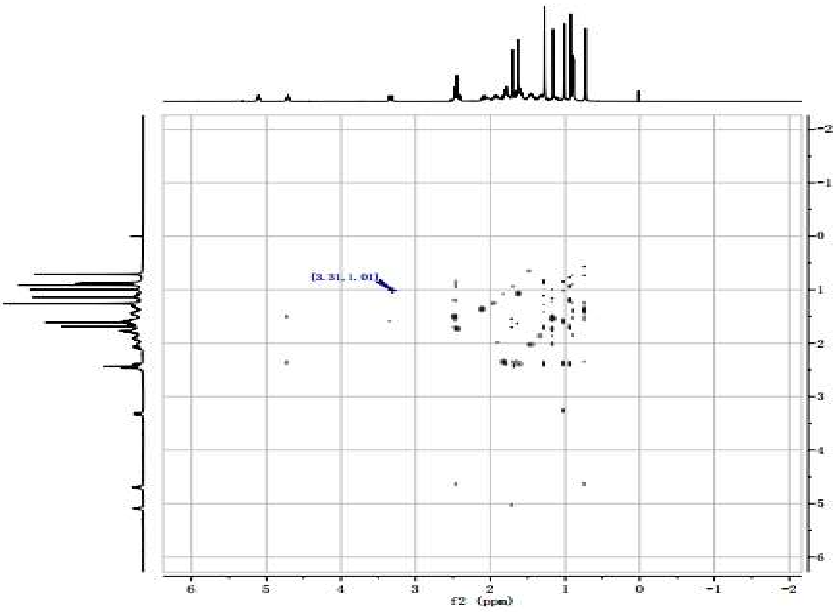

Figure 2-5. The 2D-NOESY of eupha-8,23-diene-3β,11β-diol-7-one

Compound 3

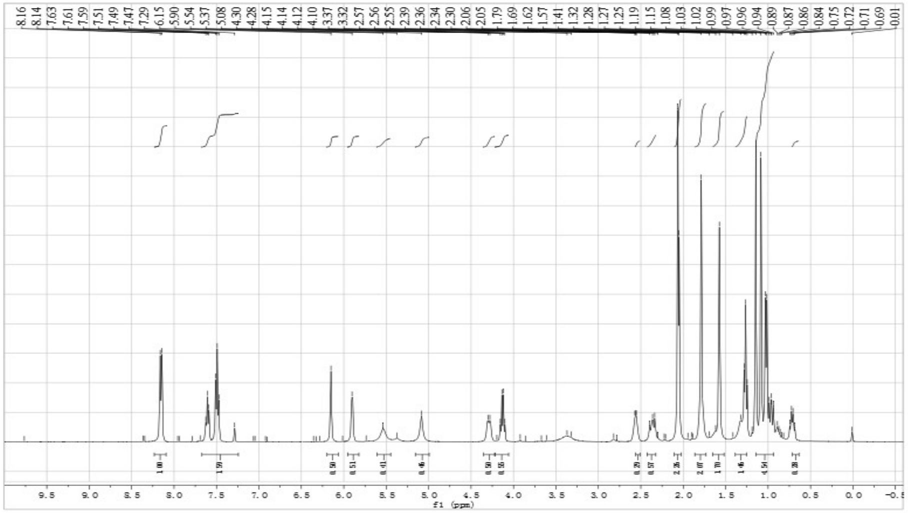

Figure 3-1. The ^{1}H-NMR of kansuiphorbin C

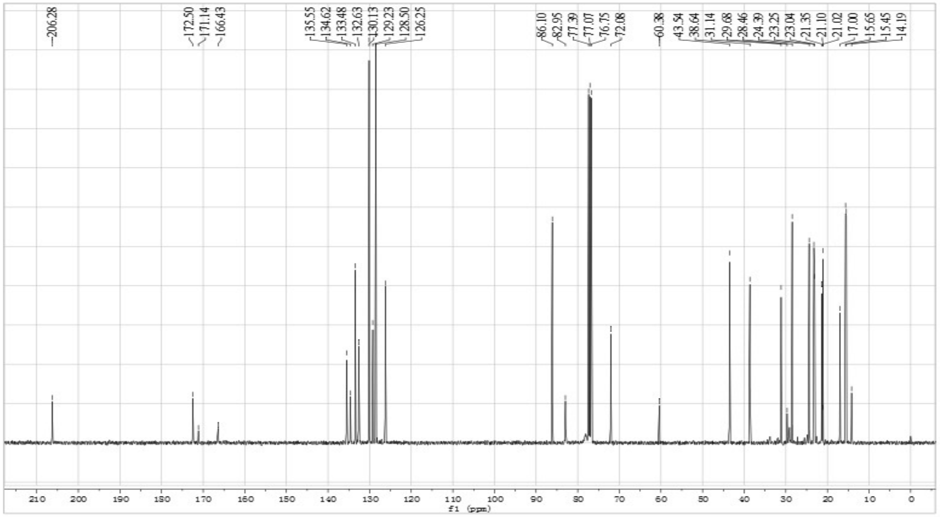

Figure 3-2. The ^{13}C-NMR of kansuiphorbin C

Compound 4

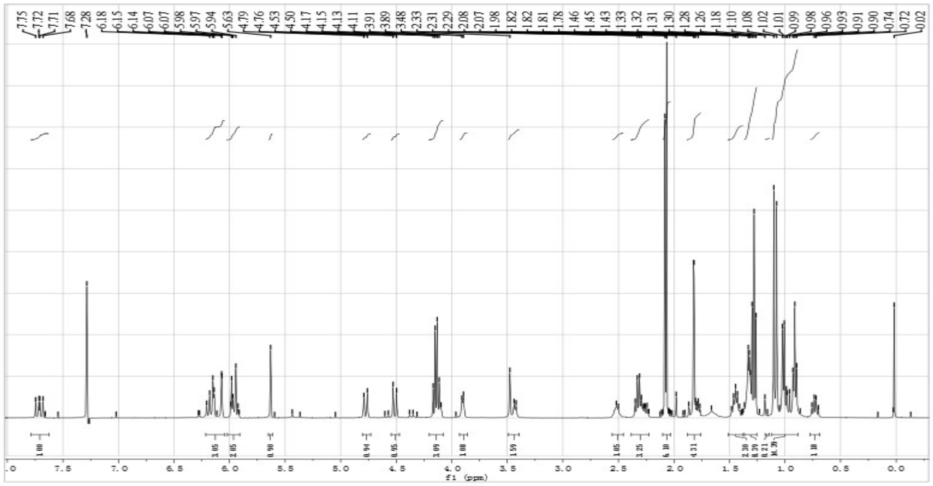

Figure 4-1. The ^{1}H-NMR of 3-*O*-(*2'E,4'Z*-decadienoyl)-20-*O*-acetylingenol

Compound 5

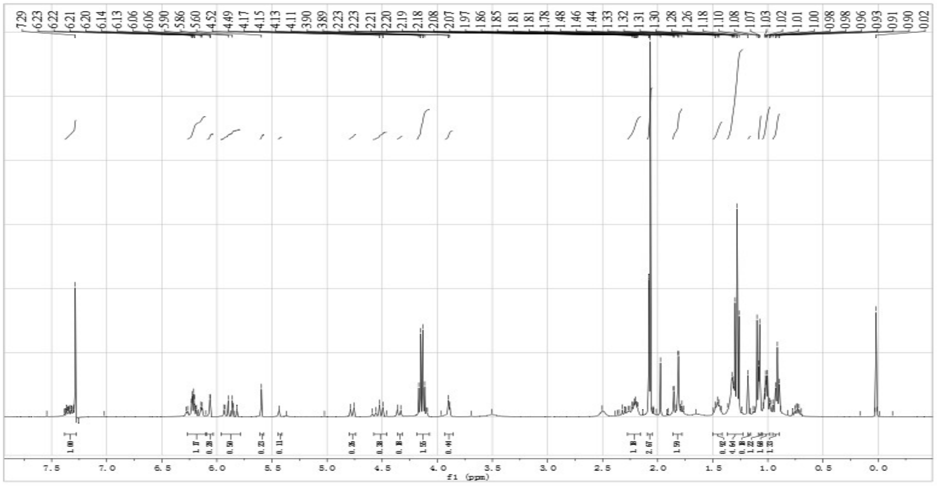

Figure 5-1. The ^{1}H-NMR of 3-*O*-(*2'E,4'E*-decadienoyl)-20-*O*-acetylingenol

Compound 6

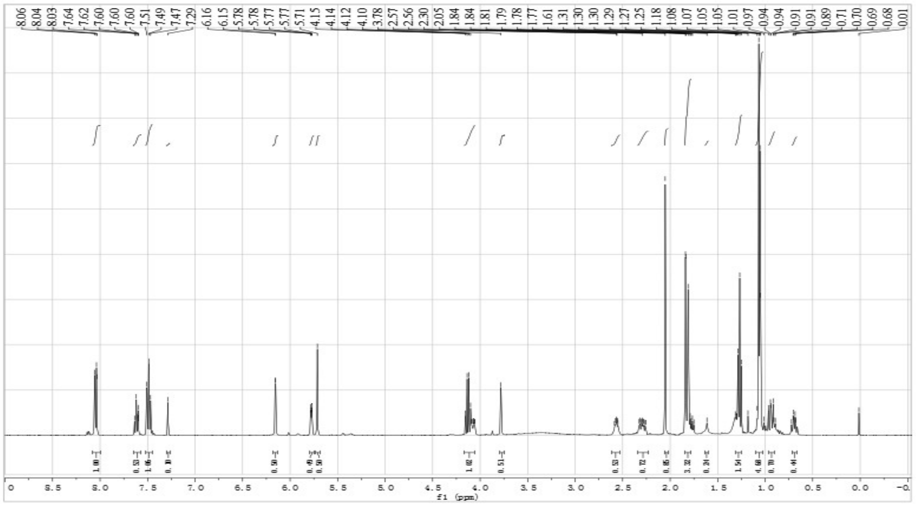

Figure 6-1. The ^{1}H-NMR of 3-*O*-benzoyl-20-deoxyingenol

Compound 7

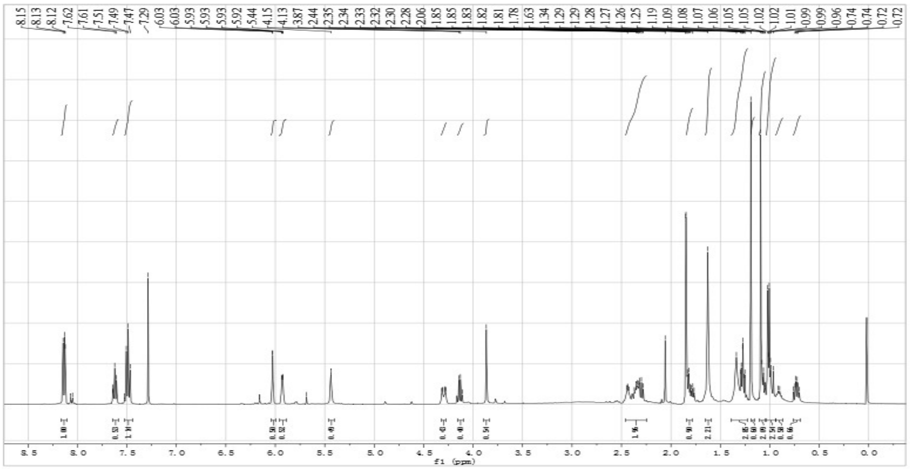

Figure 7-1. The ^{1}H-NMR of 5-*O*-benzoyl-20-deoxyingenol

Compound 8

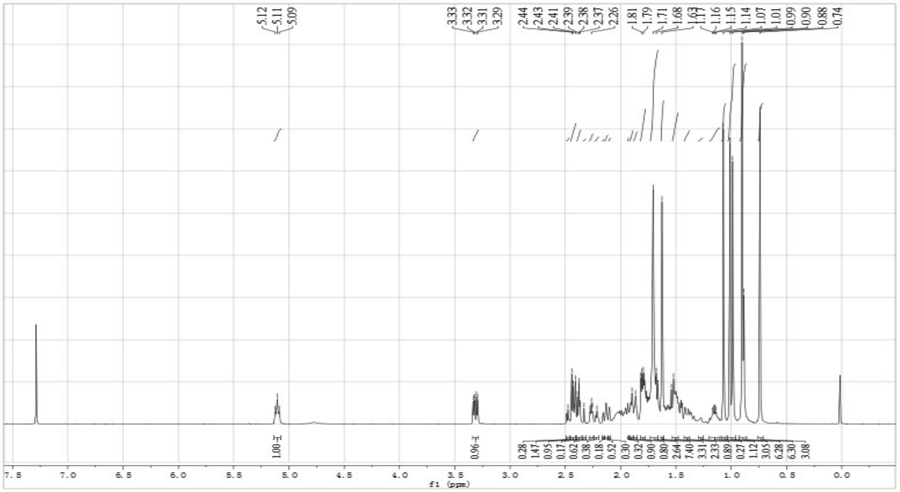

Figure 8-1. The ^{1}H-NMR of kansenone

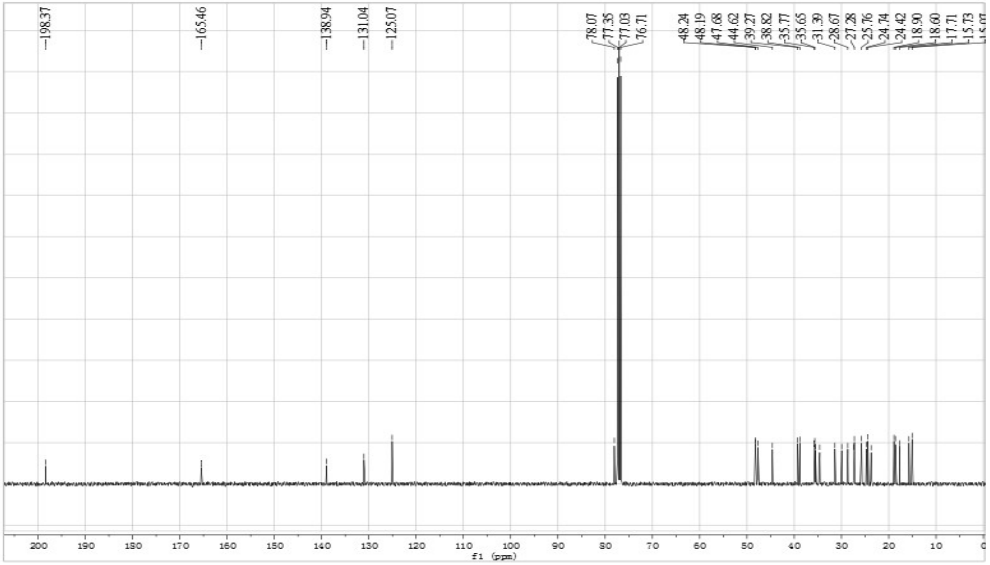

Figure 8-2. The ^{13}C-NMR of kansenone

Compound 9

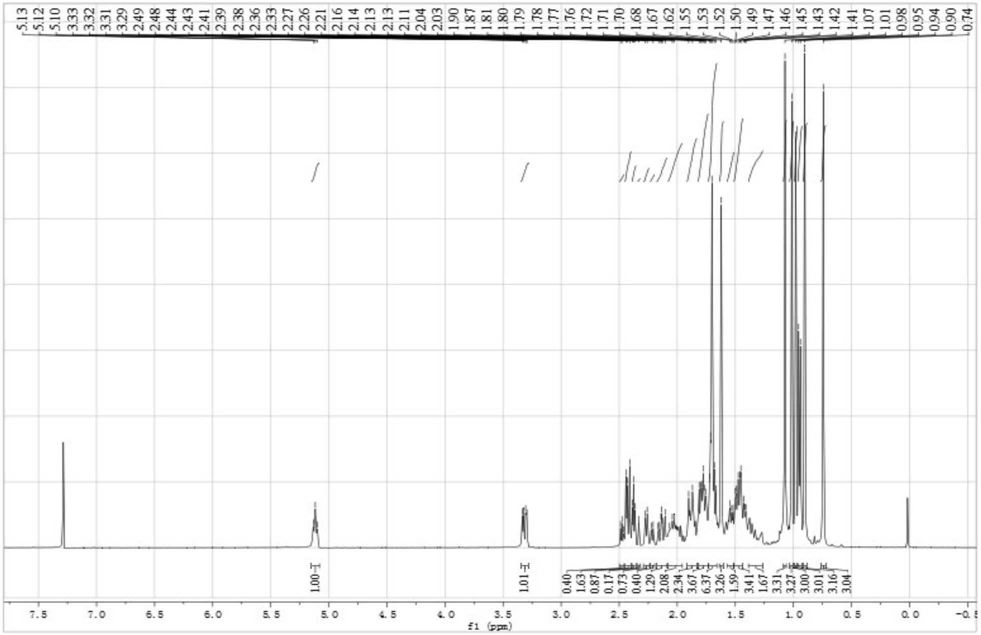

Figure 9-1. The ^{1}H-NMR of *epi*-kansenone

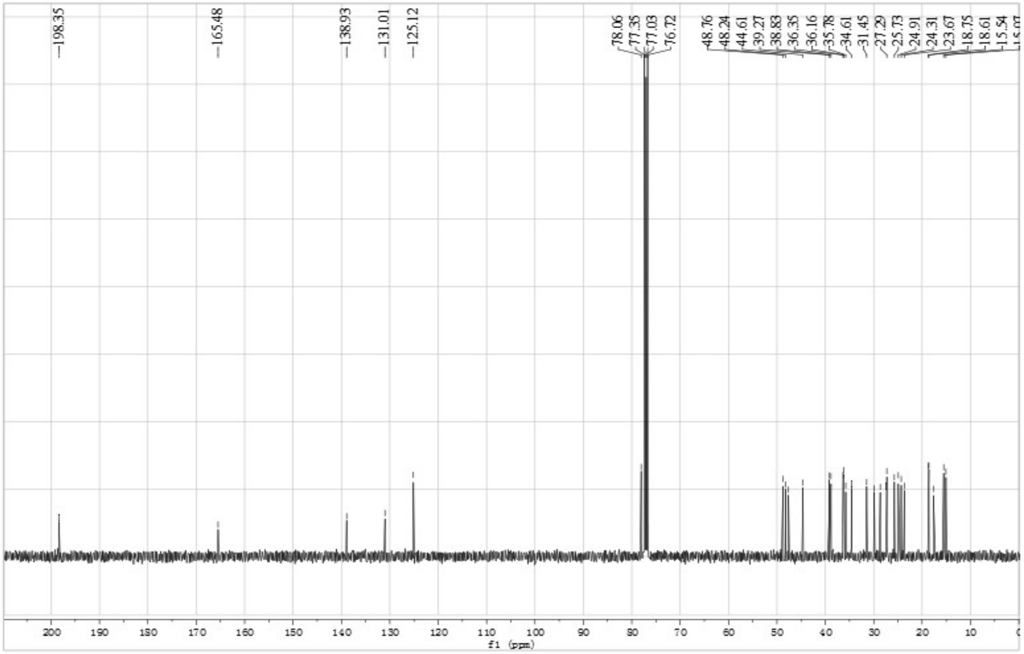

Figure 9-2. The ^{13}C-NMR of *epi*-kansenone

Compound 10

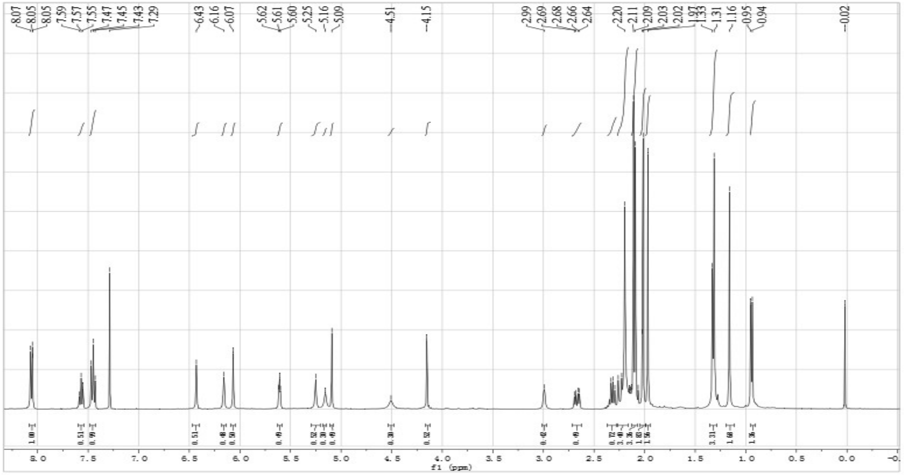

Figure 10-1. The ^{1}H-NMR of kansuinin A

Compound 11

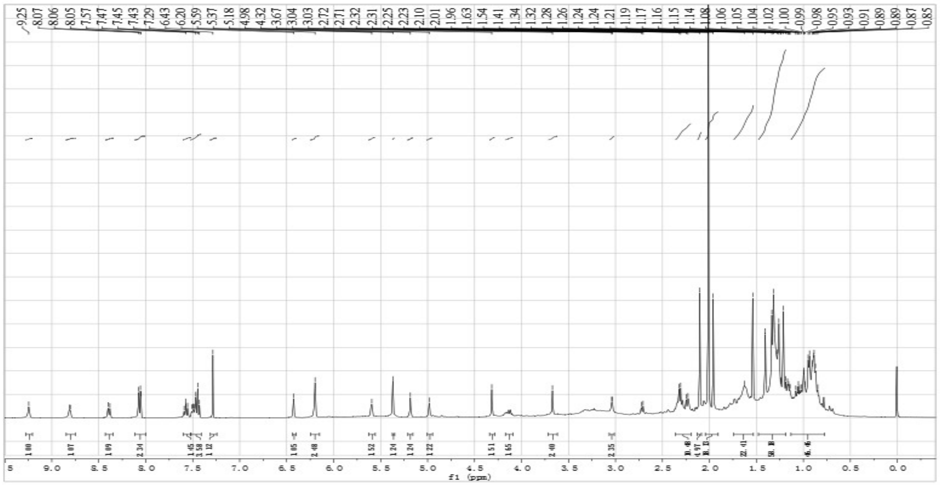

Figure 11-1. The 1H-NMR of kansuinin D

Compound 12

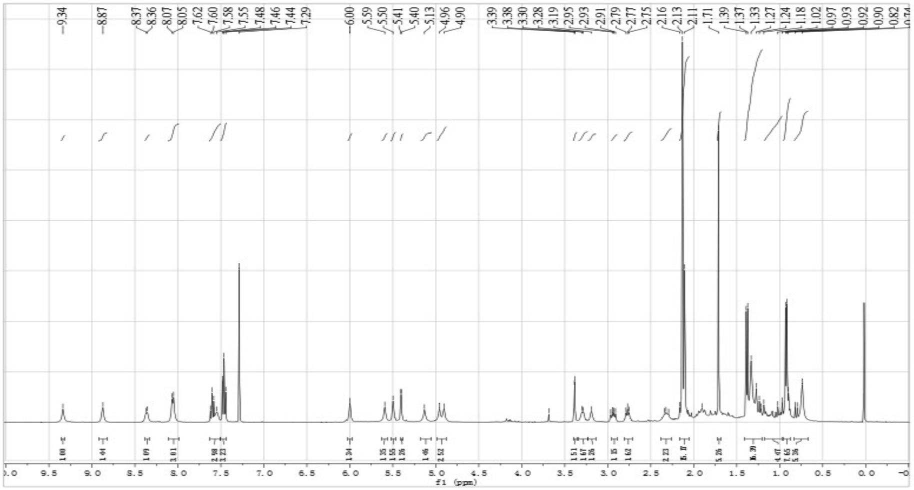

Figure 12-1. The ^{1}H-NMR of kansuinin E

Compound 13

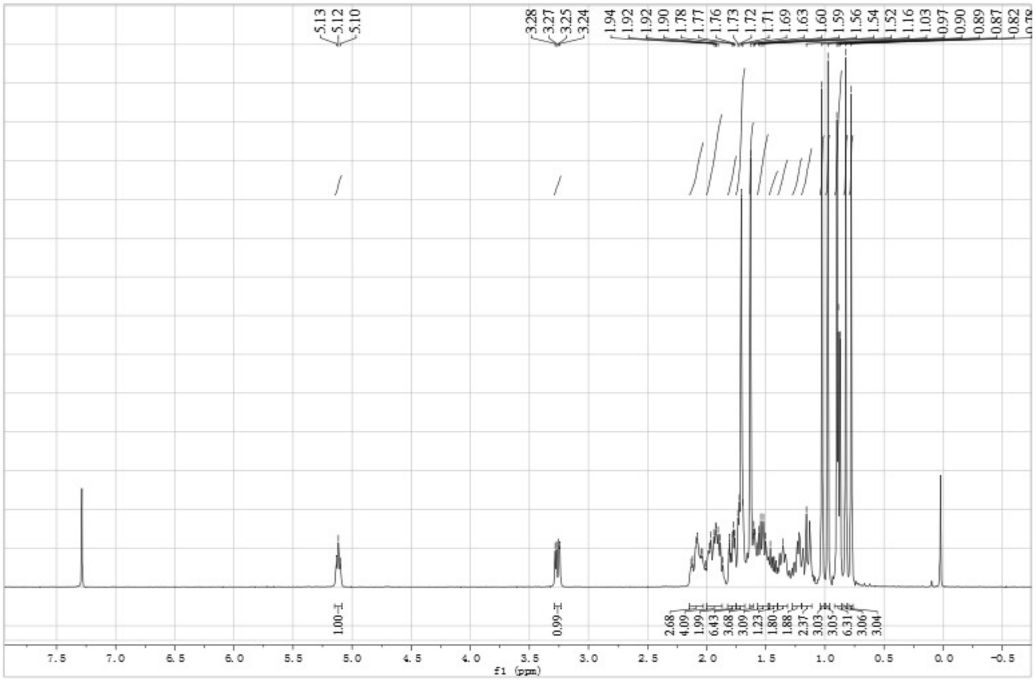

Figure 13-1. The ^{1}H-NMR of euphol

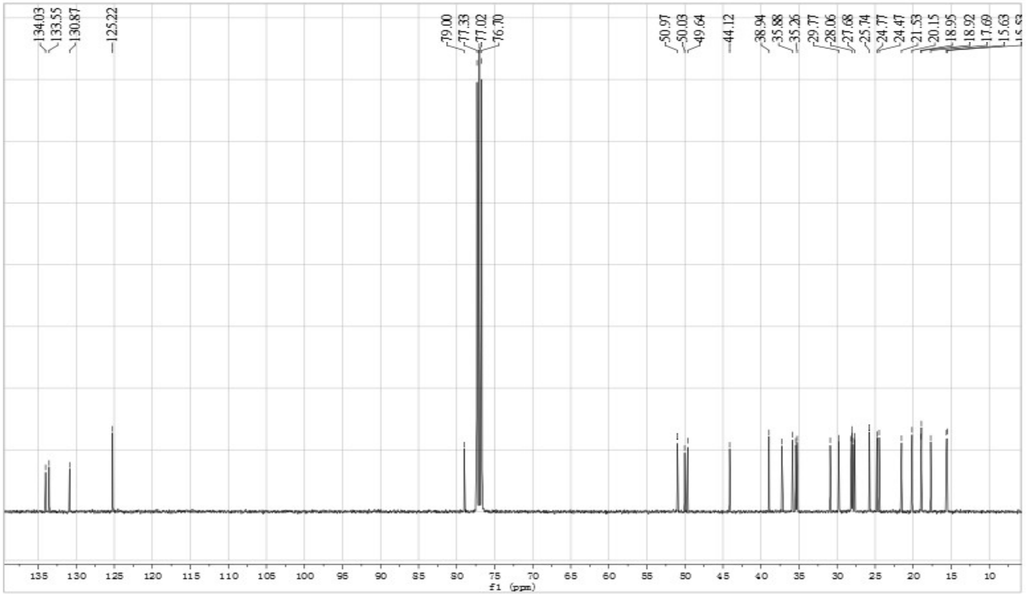

Figure 13-2. The ^{13}C-NMR of euphol

Compound 14

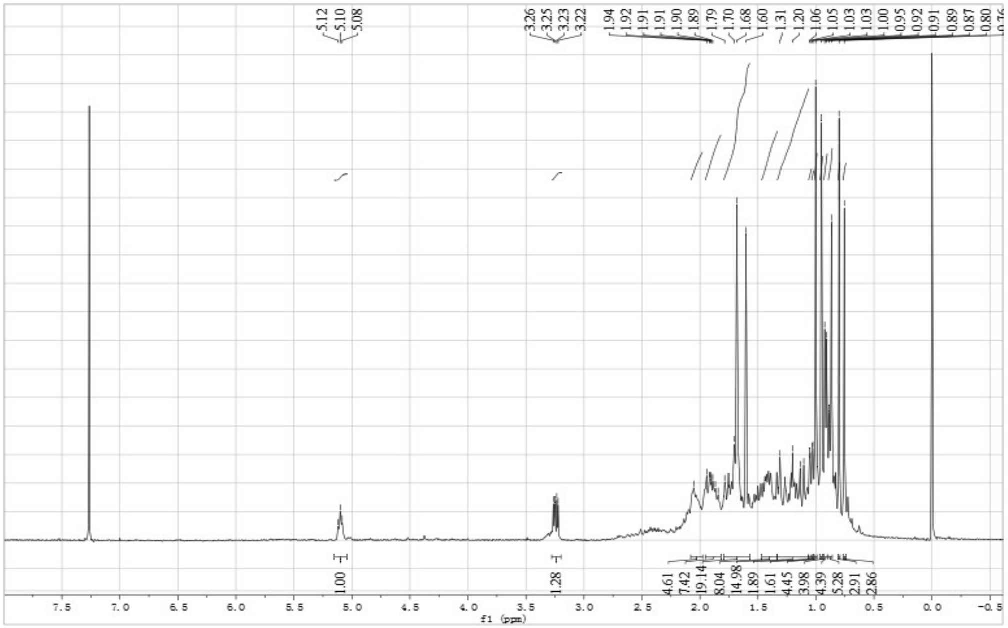

Figure 14-1. The ^{1}H-NMR of tirucallol

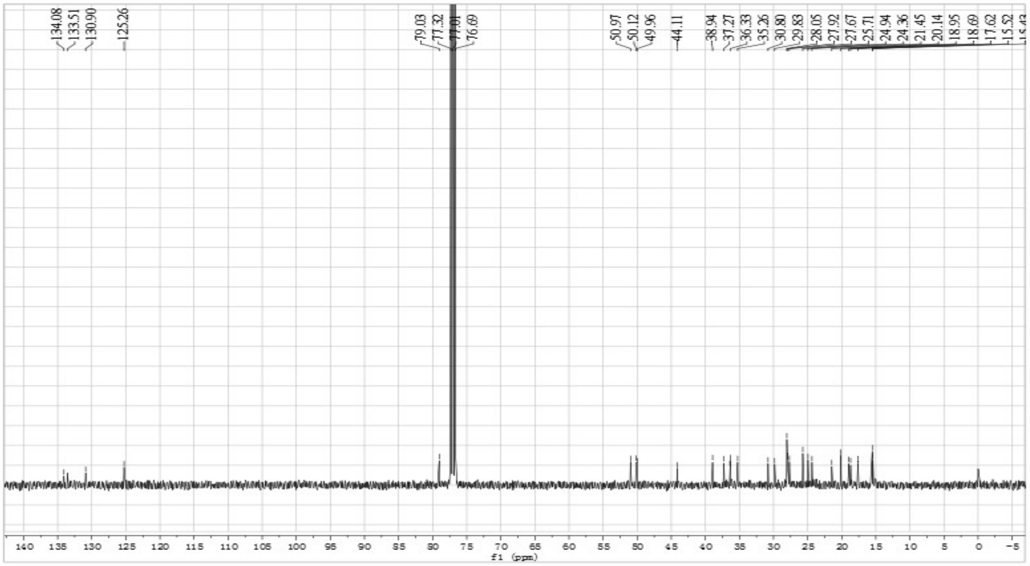

Figure 14-2. The ^{13}C-NMR of tirucallol
